# G-CSF is a key modulator of MDSC and could be a potential therapeutic target in colitis-associated colorectal cancers

**DOI:** 10.1007/s13238-015-0237-2

**Published:** 2016-01-21

**Authors:** Wenbin Li, Xinghua Zhang, Yongkang Chen, Yibin Xie, Jiancheng Liu, Qiang Feng, Yi Wang, Wei Yuan, Jie Ma

**Affiliations:** State Key Laboratory of Molecular Oncology, Cancer Hospital, Chinese Academy of Medical Sciences, Peking Union Medical College, Beijing, 100021 China; Department of Abdominal Surgical Oncology, Cancer Hospital, Chinese Academy of Medical Sciences, Peking Union Medical College, Beijing, 100021 China; Department of VIP, Cancer Hospital, Chinese Academy of Medical Sciences, Peking Union Medical College, Beijing, 100021 China

**Keywords:** inflammation, cancer, granulocyte colony-stimulating factor, myeloid derived suppressor cells

## Abstract

Granulocyte colony-stimulating factor (G-CSF) is an essential regulator of neutrophil trafficking and is highly expressed in multiple tumors. Myeloid derived suppressor cells (MDSCs) promote neoplastic progression through multiple mechanisms by immune suppression. Despite the findings of G-CSF function in colon cancer progression, the precise mechanism of G-CSF on MDSCs regulation and its blockade effects on tumor growth remains a worthy area of investigation. In this study we observed an overexpression of G-CSF in a mouse colitis-associated cancer (CAC) model, which was consistent with the accumulation of MDSCs in mouse colon tissues. Further *in vitro* studies demonstrated that G-CSF could promote MDSCs survival and activation through signal transducer and activator of transcription 3 (STAT3) signaling pathway. Moreover, compared with isotype control, anti-G-CSF mAb treatment demonstrated reduced MDSC accumulation, which led to a marked decrease in neoplasm size and number in mice. Our results indicated that G-CSF is a critical regulating molecule in the migration, proliferation and function maintenance of MDSCs, which could be a potential therapeutic target for colitis-associated cancer.

**Electronic supplementary material:**

The online version of this article (doi:10.1007/s13238-015-0237-2) contains supplementary material, which is available to authorized users.

## INTRODUCTION

Colitis-associated cancer (CAC) is a subtype of colorectal cancer (CRC), which can be originated from inflammatory bowel disease (IBD) (Grivennikov et al., 2012; Candido and Hagemann, 2013). Preceding chronic inflammation is evident in CAC development, where oxidative stress driven by pro-inflammatory cytokines induces mutations in both oncogenes and tumor suppressor genes (Vogelstein and Kinzler, 2004; Wood et al., 2007; Gallimore and Godkin, 2013). The accumulation of mutations in oncogenes and tumor suppressor genes combined with tumor microenvironment can finally lead to tumorigenesis (Wood et al., 2007).

Myeloid derived suppressor cells (MDSC), a relatively newly discovered leukocyte population induced in both cancer patients and animal models, promote neoplastic progression through multiple mechanisms by immune suppression (Gabrilovich and Nagaraj, 2009; Thevenot et al., 2014; Condamine et al., 2015). MDSC are found both systemically in the blood and locally at sites of disease (Condamine et al., 2015). In mouse models, MDSC subsets can be divided into monocytic and granulocytic subpopulations and both have been shown to be equally immunosuppressive (Youn et al., 2008). Monocytic MDSC are designated as CD11b^+^Ly6C^hi^Ly6G^−^ (or CD11b^+^Gr-1^low^), whereas granulocytic MDSC as CD11b^+^Ly6C^low^Ly6G^+^ (or CD11b^+^Gr-1^hi^) (Abrams and Waight, 2012; Filipazzi et al., 2012). Activated MDSC in pathological conditions results in over production of reactive oxygen species (ROS) and reactive nitric species (RNS), which abolish T cell mediated cytotoxicity and promote tumorigenesis (Kusmartsev et al., 2004).

Granulocyte colony-stimulating factor (G-CSF), a well-known hematopoietic cytokine regulating granulopoiesis, is an essential regulator to induce neutrophil production as well as mediate its trafficking from bone marrow to the blood (Lieschke et al., 1994). G-CSF is also necessary for progenitor cells to differentiate into granulocytic lineage, such as neutrophils, eosinophils and basophils (Natori et al., 2002). G-CSF and its receptor G-CSFR were aberrantly expressed in diverse human tumors, including gastric and colon (Morris et al., 2014), bladder (Chakraborty and Guha, 2007), pancreatic (Liongue et al., 2009), ovarian and cervical cancers (Savarese et al., 2001). High levels of tumor-associated G-CSF could promote tumor angiogenesis and metastasis and correlated with poor patient outcomes (Yokoyama et al., 2005). In addition, recent studies have demonstrated that G-CSF could increase proliferation and migration of a subset of carcinoma cells expressing CD44 (Morris et al., 2014) and accumulation of granulocytic MDSC in 4T1 mouse breast tumor model (Waight et al., 2011). Despite the findings of G-CSF function in tumor progression, the precise mechanism of G-CSF on MDSC regulation and its blockade effects on tumor growth remains a worthy area of investigation.

Here, we show that, G-CSF was overexpressed in an azoxymethane/dextran sodium sulfate (AOM/DSS) treated mouse CAC model and this overexpression was consistent with the accumulation of MDSCs in mouse colon tissues. G-CSF could not only recruit MDSC into inflamed colon tissues but also maintained their immature state, promoted their proliferation and anti-apoptosis abilities. Moreover, compared with isotype control, anti-G-CSF mAb treated mice demonstrated reduced MDSC accumulation, which led to decreased neoplasm size. This study suggested that G-CSF is a key modulator of MDSC, which might exert a potential therapeutic target in CAC immunotherapy.

## RESULTS

### G-CSF expression in colitis-associated colorectal cancer

**Figure 1:**
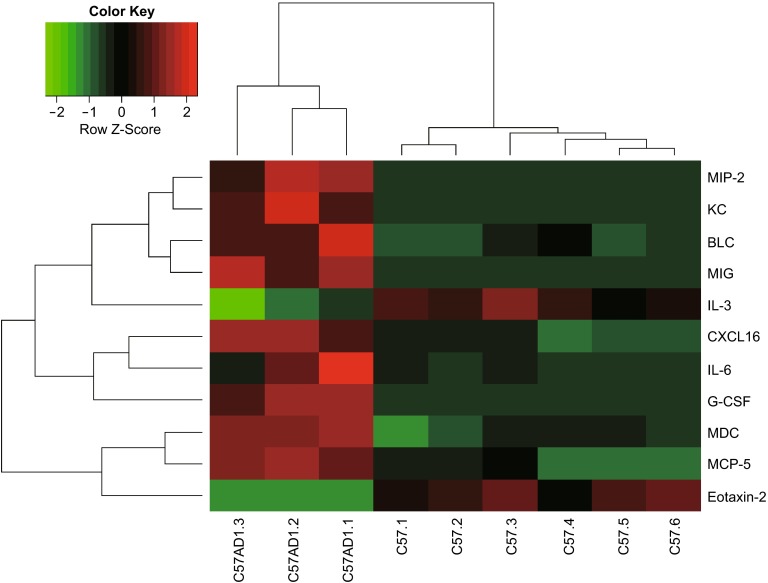
**Cytokines produced by colonic tissues from C57-AD and C57-normal mice are compared in a heat map**. Color scale and corresponding cytokine values (pg/mL) are shown

**Figure 2:**
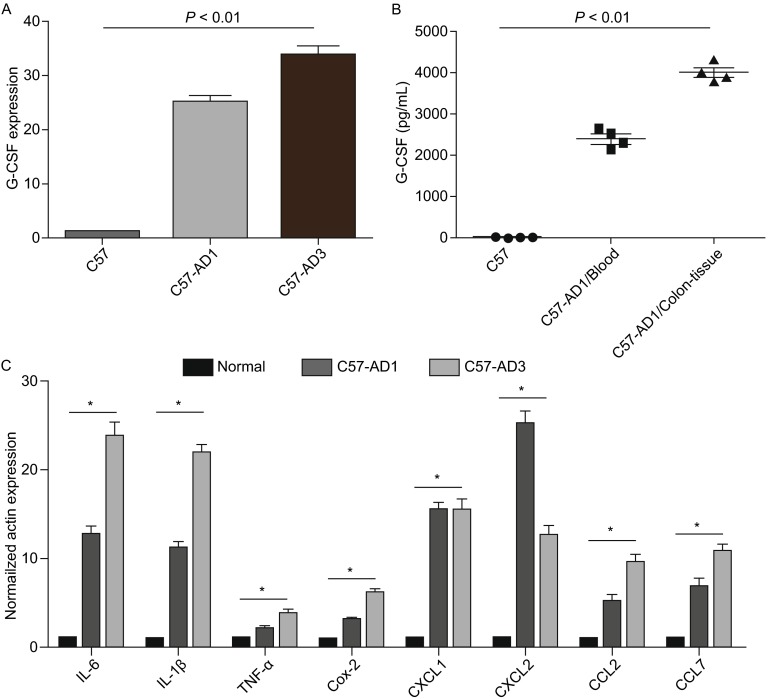
**Detection of G-CSF expression by real-time RT-PCR and ELISA assay in C57-AD and C57 normal mice**. (A) G-CSF mRNA expression in C57 and C57-AD mice. Results are given as fold increase over the mRNA level normalized β-actin and are representative of at least 3 different experiments; shown are mean ± SD from triplicate values. (B) G-CSF protein expression in colonic tissues and peripheral blood of C57 and C57-AD mice. The results represent 3 independent experiments (*n* = 4). (C) Pro-inflammatory and chemoattrants cytokines were analyzed by real-time RT-PCR. Results are given as fold increase over the mRNA level normalized to β-actin and are representative of at least 3 different experiments; shown are mean ± SD from triplicate values

Total colonic protein was extracted at stages of AD1 and AD3 and subsequently subjected to iTRAQ (isobaric Tags for Relative and Absolute Quantitation). The results showed that G-CSF was highly expressed by C57-AD compared with normal C57 mice (Fig. 1). G-CSF was elevated approximately 47-fold in C57-AD colonic tissues compared with that in normal C57 mice (see Fig. S1). In addition to the protein-chip analysis, we further examined the gene and protein expression of G-CSF in AD and normal mice. We found extremely high levels of G-CSF production not only in mRNA level in colon tissue (Fig. 2A) but also in the protein level in peripheral blood (Fig. 2B). Because a strong inflammatory status was induced in AOM/DSS model, we then examined the pro-inflammatory cytokine profiles which might play important roles in our model by using quantitative real-time PCR (qRT-PCR). Results showed that IL-6, IL-1β, TNF-α and Cox-2 mRNA were dramatically over expressed in C57-AD mice as compared to normal C57 mice. The chemoattractants of CXCL1, CXCL2, CCL2 and CCL7 for recruitment of neutrophils, MDSCs and macrophages were also observed to express at substantially elevated levels in C57-AD colonic tissues (Fig. 2C). These data indicated that G-CSF is produced at high levels in colitis associated colorectal cancer models, and may be a critical player for tumor progression.

### Properties of MDSC in colitis-associated colon tumors

**Figure 3:**
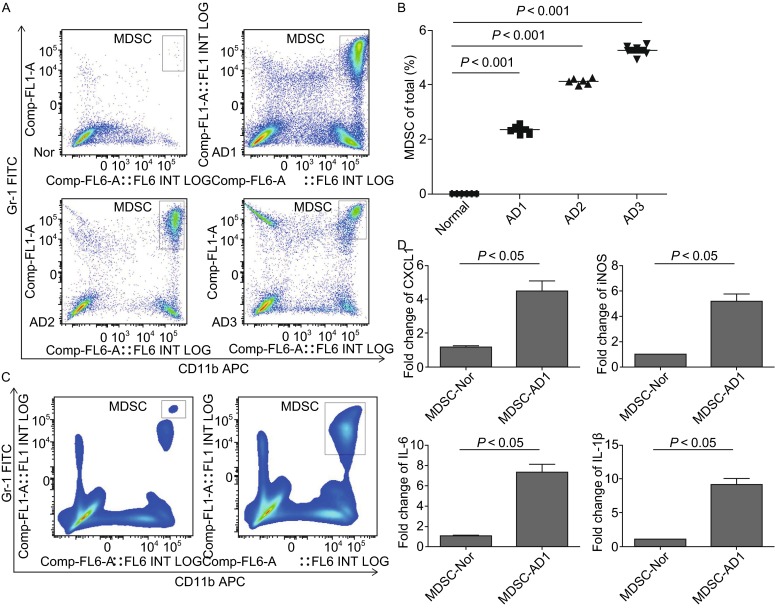
**Infiltration of MDSCs into the colonic tissues and its pro-inflammatory properities**. (A) Flow cytometry dot plot gating for MDSCs (CD11b^+^Gr-1^+^) infiltration into colon tissues along the disease development. The results represent 3 independent experiments (*n* = 4). (B) Statistical analysis of infiltrated MDSCs in the colonic tissues of C57 normal and AD mice. The results represent 3 independent experiments (*n* = 6). * represents *P* < 0.05; *P* values were obtained using two-tailed Student’s *t* tests. (C) Fluorescence-activated cell sorting of MDSCs in C57-AD mice colonic tissues and spleen of C57 mice. The left was sorted MDSCs from spleen of C57 mice and the right was sorted MDSCs from colonic tissues of C57-AD mice. (D) Total RNA from MDSCs sorted from C57 normal and C57-AD mice were analyzed by real-time RT-PCR for the expression of pro-inflammatory cytokines. Results are given as fold increase over the mRNA level normalized to β-actin and are representative of at least 3 different experiments; shown are mean ± SD from triplicate values

MDSC plays multiple roles in immune responses during diverse pathological conditions, notably chronic inflammation, infection and neoplasia. Flow cytometry analysis showed that the CD11b^+^GR-1^+^ MDSCs in the AD mice were infiltrated much more than that in the normal mice into the colonic tissues (5.283% ± 0.195% vs. 0.0128% ± 0.0028%, *P* < 0.001) (Fig. 3A and 3B). Then we isolated mouse colonic CD11b^+^GR-1^+^ MDSCs to determine the cytokine expression and found that higher expression of pro-inflammatory cytokines, such as IL-6, IL-1β and iNOS as compared to MDSC from the spleen of normal mice (Fig. 3C and 3D). This indicated that activated inflammatory MDSC in CAC model may participate in tumor formation by secretion of pro-inflammatory cytokines.

### Functional study of MDSCs regulated by G-CSF *in vitro*

**Figure 4:**
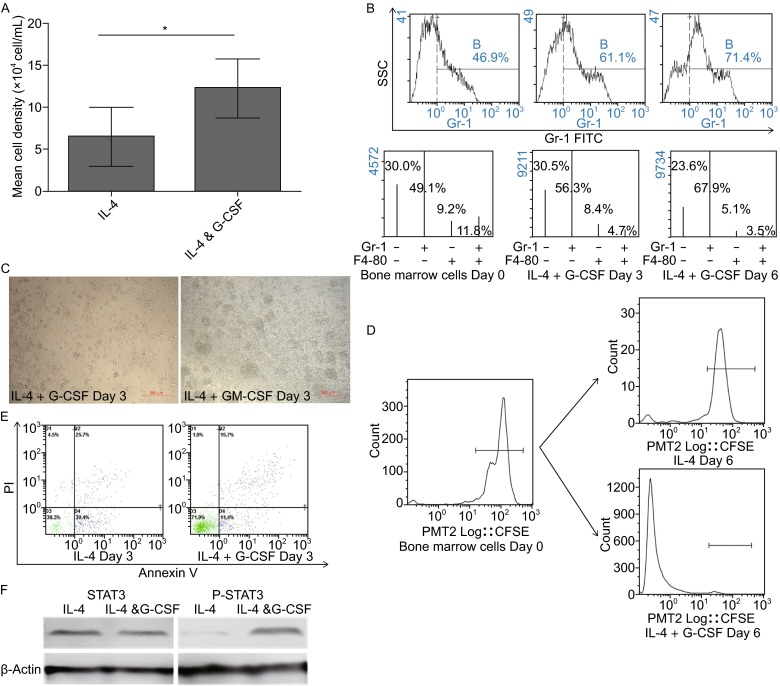
***In vitro*** **study of MDSC regulated by G-CSF**. (A) The *in vitro* study of MDSC recruited by G-CSF. MDSCs isolated from bone marrow went through a transwell membrane toward the media with IL-4 alone or IL-4/G-CSF combination for 4 h. (B–C) MDSCs isolated from bone marrow were cultured with IL-4 alone or the combination of IL-4/G-CSF for 6 days. Flow cytometric analysis of Gr-1 and F4/80 markers expressed in the cell surface of MDSCs. (D) CFSE proliferation assay. MDSCs isolated from bone marrow were incubated with CFSE previously, and then cultured with IL-4 lone or IL-4/G-CSF combination for 6 days. (E) MDSCs cultured with IL-4 lone or IL-4/G-CSF combination for 6 days, and then the apoptotic fraction was determined by using Annexin V-PE and PI fluorescences measured by a flow cytometer. (F) Western blot detection of pSTAT3 in G-CSF activated MDSCs. Equal loading is visualized by β-actin expression

There was a report recognized G-CSF as a pro-inflammatory cytokine which induces the mobilization of hematopoietic stem cells into the blood (Semerad et al., 1999). Consequently, we proposed that G-CSF in inflammatory colonic tissues might be able to recruit and maintain the function of bone-marrow derived MDSCs. In order to ascertain this hypothesis, we therefore isolated the MDSCs and performed several experiments *in vitro*. Migration experiment showed that more MDSCs went through a transwell membrane toward the media with G-CSF, which indicated that G-CSF could modulate the movement of MDSCs (Fig. 4A).

Regarding the increased MDSC number in the circulation and the evidence of chemoattractive capability of G-CSF to MDSC, we manifested that local G-CSF was released into the blood, which led to the mobilization of MDSC from bone marrow and recruitment of the cells to the colon. *In vitro* experiments also revealed that G-CSF could maintain immature status of MDSC, as the cultured bone marrow cells expressed elevated Gr-1 marker and declined F4/80 marker (Fig. 4B and 4C). Because Gr-1 was a prominent cell surface marker for mouse MDSC differentiation (Li et al., 2004) and F4/80 was for mouse macrophage differentiation (Gordon and Taylor, 2005), this flow cytometric analysis demonstrated that G-CSF could drive bone marrow cells into MDSC differentiation. Then, we performed the CFSE experiments to ascertain whether recombinant murine G-CSF could promote MDSC proliferation and found that MDSC cultured with IL-4 and rmG-CSF could induce cell proliferation compared with cells with IL-4 alone (Fig. 4D). Annexin-V PE and PI anti-apoptosis assay also revealed that G-CSF could induce the anti-apoptotic effects of MDSC (Fig. 4E). PARP (poly (ADP-ribose) polymerase) is a substrate of caspase-3 and -7 and its cleavage is one of the well-known markers for the activation of downstream signals during the apoptosis. We performed the Western blot analysis and found that the cleavage of PARP was not seen in G-CSF treated group. Because signal transducer and activator of transcription 3 (STAT3) could promote MDSCs survival and the activation of MDSCs, we performed the Western blot analysis and found that phosphorylated STAT3 was indeed over expressed in G-CSF stimulated MDSCs (Fig. 4F). Taken together, these data suggested that local G-CSF not only recruited MDSCs but also maintained their immature status, activated their proliferation and anti-apoptosis abilities.

### Anti G-CSF therapy reduce MDSC infiltration and cancer formation

**Figure 5:**
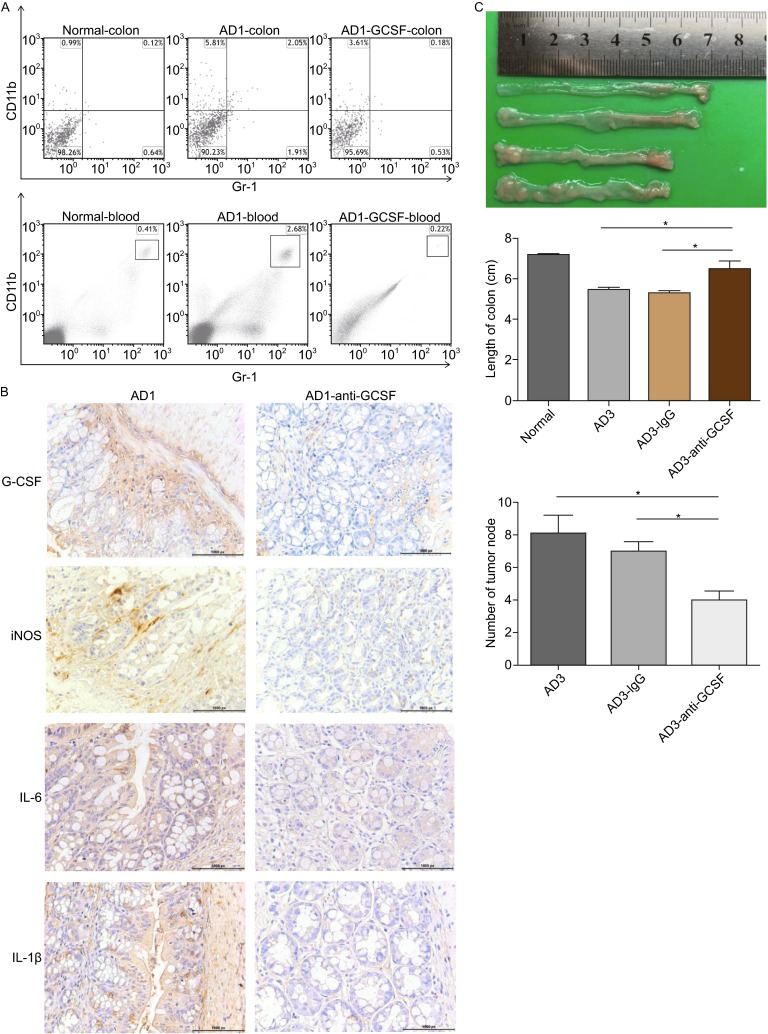
**Anti-G-CSF treatment reduces MDSC infiltration and cancer formation**. (A) The percentage of MDSCs in the colon or peripheral blood of anti-G-CSF mAb treated mice compared with control animal measured by flow cytometry. The results represent 3 independent experiments (*n* = 8). (B) The appearance of colon from wild type mice, AD3 mice treated with anti-G-CSF mAb, AD3 mice treated with rabbit IgG and control mice (from top to bottom) (up). The length of colon from the four groups mentioned above (from left to right) (middle). Number of colonic tumor node from AD3 mice treated with anti-G-CSF mAb, AD3 mice treated with rabbit IgG and control mice (from left to right) (below). The results represent 3 independent experiments (*n* = 3). * represents *P* < 0.05; *P* values were obtained using two-tailed Student's *t* tests. (C) The pro-inflammatory cytokine iNOS (up), IL-6 (middle), IL-1β (below)expression in colon after treatment with anti-G-CSF mAb (right) compared to control mice (left) by IHC. The results represent 3 independent experiments (*n* = 3)

The increased G-CSF expression and its stimulatory role in MDSCs activation prompted us to examine its therapeutic potential of anti-G-CSF treatment for CRC. After treatment with anti-G-CSF mAb, MDSC in either colon or peripheral blood was markedly reduced in comparison with control animal (Fig. 5A). The protein expression of G-CSF in colonic tissue was effectively inhibited by anti-mouse G-CSF antibody (Fig. 5B). In agreement with the reduction of MDSCs, the reduced expression of the pro-inflammatory cytokines (iNOS, IL-6, IL-1β) was observed in colon after the treatment with anti-G-CSF mAb compared to control mice by IHC. The inflammation had been controlled by anti-G-CSF treatment, which reflected in the length of the colon (Fig. 5C). Furthermore, anti-G-CSF treatment suppressed the tumor growth with only half detectable tumor node formation in the colon, suggesting an important pro-tumorigenic role of MDSC during CAC development. These data indicated that G-CSF exerted a significant impact on pro-tumor function of MDSC and anti-G-CSF therapy inhibited CAC development.

## DISCUSSION

G-CSF, the principal hematopoietic cytokine regulating granulopoiesis, is widely used for the treatment of neutropenia in a variety of clinical settings. In addition to its ability to induce neutrophil production, it is now widely accepted for its function to mobilize neutrophils from bone marrow to peripheral blood and tissues (Semerad et al., 2002). MDSCs are a rather heterogeneous subset of myeloid cells composed of immature and progenitor cells as well as mature cells either belonging to the mononuclear or the polymorphonuclear type (Gabrilovich and Nagaraj, 2009). In fact, both MDSCs and neutrophils have a common progenitor cell and granulocytic MDSCs share many similar features with neturophils (Youn et al., 2008). Despite the regulating function of G-CSF on neutrophils, it has been largely overlooked for a role in MDSC regulation and the tumor microenvironment. Our studies demonstrated that G-CSF has critical roles in MDSCs migration, proliferation and function maintenance. In addition, we further found that anti-G-CSF treatment was effective in reducing tumor burden and also inhibiting MDSCs infiltration into tumor tissues in a highly reproducible colitis-associated mouse model. Our study indicated that G-CSF may be a critical regulating molecule in CAC-associated cancer and could be a potential therapeutic target.

In mice, MDSCs are characterized by co-expression of the myeloid-cell lineage differentiation antigen Gr-1 and CD11b (Li et al., 2004). Normal mouse bone marrow contains 20%–30% of cells with this phenotype, but these cells make up only a small proportion (2%–4%) of spleen cells and are absent from the lymph nodes (Bronte et al., 2000). Our study ascertained that G-CSF could induce the differentiation of bone marrow cells into MDSCs *in vitro*. This was in accordance with the findings from previous studies, which indicated that treatment of tumor-bearing mice with recombinant G-CSF could induce the accumulation of MDSCs *in vivo* (Waight et al., 2011). In addition, the transwell migration assay of our experiment also demonstrated that G-CSF could mobilize MDSC and this indicated that G-CSF may be one of various cytokines in recruiting MDSCs from bone marrow to peripheral blood. In addition to G-CSF in MDSC recruitment from bone marrow, other studies have also demonstrated that chemokines CXCL1 and CXCL2 could promote the recruitment of MDSCs to the spleen during infection and inflammation (von Vietinghoff et al., 2010). Another cytokine stimulating factor, granulocyte-macrophage CSF (GM-CSF), exerts an important role in splenic recruitment of MDSCs in tumor bearing-mice (Bayne et al., 2012). In addition to MDSCs recruitment, G-CSF could also maintain MDSCs immature properities and promote its proliferation and anti-apoptosis abilities. Waight J.D, et al. has also demonstrated that G-CSF protein alone was sufficient to generate granulocytic-like MDSC, which strongly recapitulated phenotypic, functional and molecular characteristics observed with tumor-induced granulocytic MDSC (Waight et al., 2011).

Another existing finding of our study revealed that MDSC isolated from AOM/DSS mice could secret pro-inflammatory cytokines, such as IL-6, IL-1β and iNOS. IL-6, an important pro-inflammatory cytokines in infection and tumor, could induce differentiation of MDSC both *in vitro* and *in vivo* (Bunt et al., 2007). Previous studies have also shown that IL-6 could trigger signaling pathways in MDSCs that converge on signal transducer and activator of transcription 3 (STAT3) (Kortylewski et al., 2005; Nefedova et al., 2005). These transcription factors promote survival and activation of MDSCs, which leads to the upregulation of arginase 1 and inducible nitric oxide synthase (iNOS) (Kusmartsev et al., 2004). Our studies have also indicated the activation of MDSCs by protein expression of phosphorylate STAT3.

Several other reports also demonstrated that G-CSF expression in various solid tumors could promote tumor growth and metastasis (Morris et al., 2014). Consequently, through G-CSF blockade, we found that anti-G-CSF treatment suppressed the tumor growth as well as reduced infiltration of MDSCs in the colon. This was in accordance with previous findings that anti-G-CSF treatments appear to be very effective at reducing the number and size of neoplasms in the AOM/DSS mouse model of CRC (Morris et al., 2015). Once G-CSF was blockaded, both the MDSCs infiltration and the expression of pro-inflammatory cytokines were markedly reduced in either colon or peripheral blood.

Taken together, our findings provide evidence that G-CSF has critical roles in MDSCs migration, proliferation and function maintenance. *In vitro* studies have also demonstrated that G-CSF could promote survival and activation of MDSCs through STAT3 signaling pathway. In addition, anti-G-CSF treatment suppressed the tumor growth as well as reduced infiltration of MDSCs in the AOM/DSS mouse model of CRC. Our results indicated that G-CSF may be a critical regulating molecule in CAC and could be a potential therapeutic target.

## MATERIALS AND METHODS

### Animal experiments

Azoxymethane (AOM, MW: 74) and Dextran sodium sulfate (DSS, MW: 36,000–50,000) were purchased from Sigma-Aldrich and MP Biochemicals Inc. USA, respectively. Pathogen free 8–10 week old female wild type C57/BL6 mice and SCID mice with a C57/BL6 genetic background, were housed under specific pathogen free conditions with free access to food and water during the course of experiments. Mice were injected with a single intraperitoneal administration of AOM dissolved in physiological saline with a dose of 12.5 mg/kg body weight. Ten days later, 2.5% DSS water was given in the drinking water for 5 days, followed by 14 days of regular water. Mice were treated with 3 cycles of DSS water. At each end point of DSS water, mice fed with DSS water were defined as AD1, AD2 and AD3, respectively. Body weight was measured every week, and the animals were sacrificed at the indicated time intervals for macroscopic inspection, histological analysis, and total RNA extraction.

### Protein-chip assay

Colon tissues protein chip of cytokines and chemokines were tested by Ray-Biotech mouse protein array QAM-INT-1-2 (Capital Bio, Beijing, China) and QAM-CHE-1-2 (Capital Bio, Beijing, China) respectively. Briefly, the printed chips were blocked with 30 μL blocking solution (PBS containing 5% *w*/*v* non-fat milk)/well for 1 h at room temperature. After that, standard protein, blank control and serum samples were added in different wells for 45 min at room temperature followed by 1-h incubation with detection antibody. Finally, diluted streptavidin-Cy3 (Sigma, Missouri, USA) was added for 1 h incubation at room temperature. Extensive washing with 0.05% PBS-Tween 20 followed each incubation step to reduce nonspecific binding. The chip images were obtained by LuxScanTM 10 K Microarray Scanner (CapitalBio, Beijing, China) using Cy3 settings. The scannerhe scanner Cy3 settings. TLuxScan 3.0.0720, was used to quantify the spot fluorescence intensity from the scanned images.

### Immune cell isolation

Immune cells were isolated from colon tissues, peripheral blood, bone marrow and spleen by flow sorting. Single-cell suspensions from colon tissue dissection were prepared by manual mincing using scalpel followed by enzymatic digestion for 40 min at 37°C by Collagenase A 2.0 mg/mL (Roche, Canada) and DNase I 100U/mL (Roche, Canada) dissolved in DMEM (Gibco, USA) under continuous stirring. Digestion mixtures were resuspended by DMEM containing 10% FBS and then filtered through 70 μm nylon strainers (Falcon, BD biosciences, USA). Immune cells from peripheral blood,bone marrow and spleen were obtained by Ficoll-Hypaque (TBD science, China) density centrifugation 2000 rpm for 20 min.

### Flow cytometric analysis

Cells were incubated for 10 min at 4°C with rat anti-mouse CD16/CD32 mAb (1:200, Quantobio, China) at a 1:100 dilution in PBS containing 2% of FBS (Sigma, USA) to prevent nonspecific antibody binding. Subsequently, cells were washed twice in PBS/FBS and incubated for 30 min with 100 μL of each of the following fluorophore-conjugated anti-mouse antibodies: CD11b-APC (M1/70), CD19-APC (MB19-1), CD45-PE-cy7 (30-F11), F4/80-PE (BM8) and Gr-1-FITC (RB6-8C5) (all from eBioscience, USA). Antibodies were used at 1:100 dilution in PBS containing 2% FBS. Data acquisition was performed on a FACS Calibur using CellQuestPro software (BD Biosciences, USA) and analysis carried out using FlowJo software program (Tree Star Inc, USA).

### Quantitative real time PCR

Total RNA was extracted from 500,000 FACS sorted CD45^+^CD11b^+^Gr1^+^ cells by using an RNeasy Mini Kit (Qiagen). The cDNAs were synthesized by using Superscript III First-Strand Synthesis (Invitrogen). Primers specific for β-actin, IL-6, G-CSF, NOS2, IL-1β (Invitrogen) were used and relative gene expression determined by using RT Real-Time SYBR Green/ROX PCR master mix (Takara, Japan) on an LightCycler 480 quantitative PCR machine (Roche). The comparative threshold cycle method was employed to calculate the changes in gene expression, which was normalized to both β-actin as reference genes. Samples were assayed from at least three independent experiments per category. The primers of genes were listed below:

**Table:** 

Gene name	Forward primer	Reverse primer
IL-6	5′-GCTAAGGACCAAGACCATCCAAT-3′	5′-GGCATAACGCACTAGGTTTGC-3′
G-CSF	5′-TGCTTAAGTCCCTGGAGCAA-3′	5′-AGCTTGTAGGTGGCACACAA-3′
NOS2	5′-CACCTTGGAGTTCACCCAGT-3′	5′-ACCACTCGTACTTGGGATGC-3′
IL-β	5′-GCCCATCCTCTGTGACTCAT-3′	5′-AGGCCACAGGTATTTTGTCG-3′
β-actin	5′-AGCCATGTACGTAGCCATCC-3′	5′-CTCTCAGCTGTGGTGGTGGA-3′

### ELISA assay

Various G-CSF concentrations with conditioned medium were assayed by using Ready-Set-Go ELISA kit (R&D systems) as described by the manufacturer. Optical density was measured at 450 nm with wavelength correction set to 540 nm on a SpectraMax 340 spectrophotomoter (Molecular Devices).

### MDSC migration assay

MDSCs were obtained by flow cytometric sorting. MDSCs were washed with PBS twice and resuspended with 5 × 10^5^/mL in a DMEM with 10% FBS, 10 ng/mL rmIL-4 (Peprotech, USA). Transwell chambers (Corning, USA) were placed in a 24-well plate, the lower chamber was filled with DMEM with 10% FBS, 10 ng/mL rmIL-4 with/without 100 ng/mL rmG-CSF (Peprotech, USA), and the upper chamber was filled with MDSCs. After 4 h, cells were harvested in the lower chamber and counted by the cells counts.

### Bone marrow cells proliferation assay

Cells were resuspended at 1~2 × 10^6^/mL in a minimum volume of 1 mL PBS and incubated with carboxyfluorescein succinimidyl amino ester (CFSE, 5 mmol/L) at 37°C for 10 min with constant swirling. The reaction was then quenched with media containing 10% FCS, washed with PBS for two times. Cells labeled with CFSE were cultured in 96-well plates (1 × 10^5^ cells/well) and stimulated with 100 ng/mL G-CSF plus 10 ng/mL IL-4 or 10 ng/mL IL-4 alone, respectively. After incubation for 6 days, cells were harvested and analyzed by using FACS Calibur (BD).

### Cell apoptotic assay

Bone marrow cells were cultured with 100 ng/mL G-CSF plus 10 ng/mL IL-4 or 10 ng/mL IL-4 alone, respectively. After 6 days, cells were harvested and tested for apoptosis. The apoptotic fraction was determined by using Annexin V-PE apoptosis kit (Sounthern Biotech, Birmingherm, AL, USA) for 15 min at 4°C in dark in accordance with the manufacturer’s instructions, followed by adding with Propidine iodide (PI). Annexin-V PE and PI fluorescences were measured by using a flow cytometer.

### Western blot analysis

Cells were lysed in 1× SDS-PAGE sample buffer, and the total protein was quantified using the BCA protein assay reagent (Thermo Fisher, Grand Island, NY, USA). The cell lysates were loaded onto a 10% SDS-PAGE gel and separated and then electrophoretically transferred to a polyvinylidene fluoride membrane. The membrane was blocked in a 5% skim milk suspension for 1 h at room temperature prior to an overnight incubation at 4°C with one of the following primary antibodies: anti-poly (ADP-ribose) polymerase (PARP) (9542, Cell Signaling Technology, Danvers, MA, USA), anti-phospho-Stat3 (Tyr 705) (D3A7, Cell Signaling Technology, Danvers, MA, USA). The membrane was blocked in a 5% skim milk suspension for 1 h at room temperature prior to an overnight incubation at SA), anti-phospho-Stat3 (Tyr 705) (D3A7, Cell Signaling Technology, Danvers, MA, USA). The membrane was subsequently incubated with the anti-rabbit or anti-mouse HRP-IgG (Santa Cruz Biotechnology) secondary antibody for 1 h at room temperature. Chemiluminescence was detected with an ECL blot detection system (Santa Cruz Biotechnology).

### G-CSF treatment

Pathogen free 8- to 10-week old female WT C57/BL6 mice and SCID mice on a C57/BL6 genetic background, were housed under specific pathogen-free conditions with free access to food and water during the course of experiments. Mice were injected with a single intraperitoneal administration of AOM dissolved in physiological saline of a dose of 12.5 mg/kg body weight. Ten days later, 2.5% DSS water was given in the drinking water for 5 days, followed by 14 days of regular water. Mice were treated with 3 cycles of DSS water. G-CSF was neutralized through IV injection at a dose of 10 mg/kg of G-CSF mAb (Cat No. 500-P69, Peprotech) 1 h before each DSS feeding. Mice were sacrificed at AD1 and AD3. Bone marrows, spleens, peripheral blood, colon tissues were collected and measured as described previously.

## Electronic supplementary material

Supplementary material 1 (PDF 183 kb)
