# Supplementary material for: G-CSF is a key modulator of MDSC and could be a potential therapeutic target in colitis-associated colorectal cancers
Source: Protein Cell. 2016 Jan 21;7(2):130–40. doi: 10.1007/s13238-015-0237-2 (PMC4742385; doi:10.1007/s13238-015-0237-2)
Supplement: Supplementary file 1 — Supplementary material 1 (PDF 183 kb) [file 13238_2015_237_MOESM1_ESM.pdf]

Protein chip assay in C57 normal and AD mice

	C57-AD1/Normal	C57-AD3/normal
G-CSF	47.3	3.2
MIG（CXCL9）	4.7	6.7
I-TAC（CXCL11）	2.2	3.8
IL-17	1.2	1.0
IL-10	1.0	0.7
IL-1β	0.7	0.8
MIP-3α（CCL20）	1.4	2.6
KC（CXCL1）	6.7	18.0
MIP-2（cxcl2）	10.0	2.5
TNFα	0.8	0.9
IFN-γ	0.7	0.8
MDC（CCL22）	6.7	4.0
BLC（CXCL13）	4.4	3.9
IL-6	3.5	0.9
IL-23	0.6	0.8
IL-4	1.2	0.7

Supplementary Figure 1. Protein-chip assay in C57-normal and C57-AD mice.

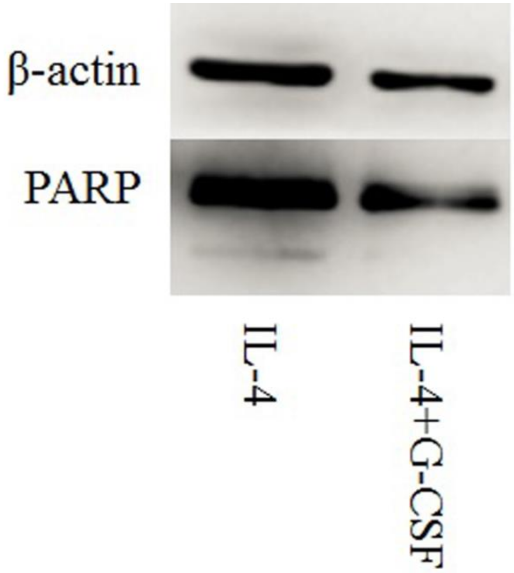

Supplementary Figure 2. Anti-apoptotic effect of G-CSF treated MDSCs. PARP cleavage was detected in IL-4 treated cells but not in IL-4 plus G-CSF treated cells.
